# Clinical outcome comparison of Grade Group 1 and Grade Group 2 prostate cancer with and without cribriform architecture at the time of radical prostatectomy

**DOI:** 10.1111/his.14064

**Published:** 2020-04-21

**Authors:** Eva Hollemans, Esther I Verhoef, Chris H Bangma, John Rietbergen, Monique J Roobol, Jozien Helleman, Geert J L H van Leenders

**Affiliations:** ^1^ Department of Pathology Erasmus MC University Medical Centre Rotterdam The Netherlands; ^2^ Department of Urology Erasmus MC University Medical Centre Rotterdam The Netherlands; ^3^ Department of Urology Franciscus Gasthuis & Vlietland Rotterdam The Netherlands

**Keywords:** cribriform, intraductal carcinoma, prognosis, prostate cancer

## Abstract

**Aims:**

Invasive cribriform and intraductal carcinoma are associated with aggressive disease in Grade Group 2 (GG2) prostate cancer patients. However, the characteristics and clinical outcome of patients with GG2 prostate cancer without cribriform architecture (GG2−) as compared with those with Grade Group 1 (GG1) prostate cancer are unknown. The aim of this study was to investigate the clinical and pathological characteristics of GG1 and GG2− prostate cancer in radical prostatectomy specimens.

**Methods and results:**

We reviewed 835 radical prostatectomy specimens for Grade Group, pT stage, surgical margin status, and the presence of cribriform architecture. Biochemical recurrence‐free survival and metastasis were used as clinical outcomes. GG1 prostate cancer was seen in 207 patients, and GG2 prostate cancer was seen in 420 patients, of whom 228 (54%) showed cribriform architecture (GG2+) and 192 (46%) did not. GG2− patients had higher prostate‐specific antigen levels (9.4 ng/ml versus 7.0 ng/ml; *P* < 0.001), more often had extraprostatic extension (36% versus 11%; *P* < 0.001) and had more positive surgical margins (27% versus 17%; *P* = 0.01) than GG1 patients. GG2− patients had shorter biochemical recurrence‐free survival (hazard ratio 2.7, 95% confidence interval 1.4–4.9; *P* = 0.002) than GG1 patients. Lymph node and distant metastasis were observed neither in GG2− nor in GG1 patients, but occurred in 22 of 228 (10%) GG2+ patients.

**Conclusion:**

In conclusion, patients with GG2− prostate cancer at radical prostatectomy have more advanced disease and shorter biochemical recurrence‐free survival than those with GG1 prostate cancer, but both groups have a very low risk of developing metastasis.

## Introduction

Active surveillance is increasingly being applied for patients with prostate cancer. Whereas most patients with biopsy Grade Group 1 (Gleason score of 3 + 3 = 6, GG1) prostate cancer are eligible for active surveillance, the inclusion of favourable Grade Group 2 (Gleason score of 3 + 4 = 7, GG2) patients with limited Gleason pattern 4 is gradually being accepted.^1^, ^2^, ^3^, ^4^, ^5^ In general, these patients have prostate‐specific antigen (PSA) levels of <10 ng/ml, present with organ‐confined disease, and have <10% Gleason pattern 4 in their diagnostic biopsies.^6^

Gleason pattern 4 prostate cancer is a heterogeneous disease encompassing various histopathological growth patterns. Invasive and/or intraductal cribriform carcinoma, both also referred to as cribriform architecture, have been identified as pathological parameters for worse outcome in both biopsy and radical prostatectomy specimens.^7^, ^8^, ^9^, ^10^, ^11^, ^12^ Cribriform architecture has been associated with advanced tumour stage, biochemical recurrence, metastasis and disease‐specific death in GG2 patients.^13^, ^14^, ^15^ Although patients with GG2 prostate cancer without cribriform architecture (GG2−) have favourable outcomes as compared with those with invasive and/or intraductal cribriform carcinoma (GG2+), it is unclear to what extent GG2− prostate cancer differs from GG1 prostate cancer.

In previous sextant biopsy studies with long‐term follow‐up, biopsy GG2− patients had similar biochemical recurrence‐free and disease‐specific survival as GG1 patients.^16^, ^17^ Therefore, it has been proposed that patients without cribriform architecture might be eligible for active surveillance.^15^, ^16^, ^17^, ^18^, ^19^, ^20^ However, prostate biopsies are subject to significant sampling errors with tumour undergrading in up to 40%, and there is low sensitivity for detection of cribriform architecture.^21^, ^22^, ^23^ Moreover, in contrast to radical prostatectomy specimens, minor high‐grade patterns are always taken into account when prostate cancer biopsies are graded. To elucidate its clinical and biological features, GG2− prostate cancer should be investigated on radical prostatectomy specimens, which excludes study bias caused by biopsy sampling artefacts. The aim of this study was to compare the clinicopathological characteristics and biochemical recurrence‐free survival of GG1 patients and GG2− patients in radical prostatectomy specimens.

## Materials and methods

### Patient Selection

In total, 854 patients who had undergone radical prostatectomy for prostate adenocarcinoma at Erasmus MC, University Medical Centre, Rotterdam, The Netherlands between 2000 and 2017 were included. Patients who had received hormonal, radiation or viral therapy (*n* = 19) prior to surgery were excluded from this study.^24^ After fixation in neutral‐buffered formalin, radical prostatectomy specimens were sectioned transversely and totally embedded for diagnostic purposes. All slides were available for pathology review. The use of tissue samples for scientific purposes was approved by the institutional Medical Research Ethics Committee (MEC‐2018‐1614).

### Pathological Evaluation

All 835 radical prostatectomy specimens were reviewed by two investigators (E.H. and G.G.L.H.v.L), who were blinded to clinical outcome. The following features were recorded: Gleason score and Grade Group according to the World Health Organization 2016 guidelines, pT stage according to the American Joint Committee on cancer TNM 8th edition, surgical margin status, Gleason pattern 3 to 5 percentages, Gleason 4 growth patterns, and the presence of intraductal carcinoma.^25^, ^26^, ^27^ Tertiary Gleason patterns occupied <5% of the total tumour area.^25^, ^27^ Intraductal carcinoma and tertiary Gleason patterns were not incorporated in the Gleason score. Invasive cribriform Gleason grade 4 was morphologically distinguished from intraductal carcinoma when it had an irregular outline, anastomosing fields beyond pre‐existing gland architecture, or extension into periprostatic fat tissue, ejaculatory ducts, or seminal vesicles. Intraductal carcinoma was morphologically identified if cribriform structures were clearly continuous with pre‐existing glands lined by normal basal epithelium, or containing corpora amylacea. When invasive cribriform carcinoma and intraductal carcinoma could not be differentiated by the use of morphological criteria alone, additional immunohistochemical staining for the presence of basal cells was performed.

### Immunohistochemistry

Four‐micrometre‐thick tissue sections were cut from selected paraffin‐embedded blocks (Superfrost Microscopic Slides; ThermoFisher Scientific, Bleiswijk, The Netherlands). Slides were deparaffinised, and rehydrated with xylene and ethanol. Endogenous peroxidase was blocked with 0.3% H_2_O_2_ in phosphate‐buffered saline, and heat‐induced antigen retrieval was accomplished by 15 min of incubation in Tris‐EDTA buffer (pH 9; Klinipath, Duiven, The Netherlands). Mouse monoclonal high molecular weight cytokeratin (clone 34BE12; 1:200; Dako, Heverlee, Belgium) diluted in normal antibody diluent (APG‐500; ScyTek Laboratories, West Logan, WV, USA) was incubated for 2 h at room temperature. Antibody visualisation was performed with the Envision kit (Dako) and slide counterstaining with haematoxylin. When basal cell staining was absent, the cribriform structure was classified as invasive carcinoma; if sporadic, scattered or continuous basal cells were identified, the growth pattern was classified as intraductal carcinoma.

### Clinical Follow‐Up

Clinical follow‐up after radical prostatectomy consisted of 6‐monthly, and later annual, monitoring of serum PSA levels. Biochemical recurrence was defined as a PSA level of ≥0.2 ng/ml measured at two separate points in time at least 3 months apart when PSA had been undetectable after surgery, or as a PSA increase of >2.0 ng/ml whenever serum PSA had not declined to zero after surgery. Postoperative lymph node and distant metastases were confirmed by biopsy or multidisciplinary consensus. Biochemical recurrence‐free survival was defined as the time in months from radical prostatectomy to biochemical recurrence.

### Statistical Analysis

Normally distributed, continuous variables were analysed by use of the independent sample Student's *t*‐test. Pearson's *χ* ^2^ test was used for categorical parameters. Missing PSA values (*n* = 27) were imputed by use of the median PSA value. Biochemical recurrence‐free survival was analysed with Cox proportional hazards regression and visualised by the use of Kaplan–Meier curves. Statistical analyses were performed with spss version 24 (IBM, Chicago, IL, USA). Results were considered to be significant when the two‐sided *P*‐value was <0.05.

## Results

### Patient Characteristics

Of 835 radical prostatectomy specimens, 207 were GG1 and 420 were GG2. The median age of these 627 patients at the time of surgery was 64.1 years [interquartile range (IQR) 59.8–67.6 years], and the median PSA level was 7.6 ng/ml (IQR 5.4–10.8 ng/ml). Pathological tumour stage was distributed as follows: 419 (66%) pT2, 173 (28%) pT3a, and 35 (6%) pT3b. Positive surgical margins were present in 177 (28%) cases. Pelvic lymph node dissection was performed in 375 (60%) patients, of whom 12 (3%) had lymph node metastasis.

### Invasive Cribriform And/Or Intraductal Carcinoma

Among GG2 patients, 228 (54%) had invasive cribriform and/or intraductal carcinoma (GG2+) and 192 (46%) did not (GG2−). GG2+ patients had higher PSA levels (12.2 ng/ml versus 9.4 ng/ml; *P* = 0.006), a higher percentage of Gleason pattern 4 (24% versus 18%; *P* < 0.001), more frequent extraprostatic extension (pT3; 52% versus 36%; *P* < 0.001), more positive surgical margins (40% versus 27%; *P* = 0.007) and more lymph node metastases (8% versus 0%; *P* = 0.001) than GG2– patients. GG2− patients presented with higher median PSA levels (9.4 ng/ml versus 7.0 ng/ml; *P* < 0.001), more frequent extraprostatic extension (36% versus 11%; *P* < 0.001) and more positive surgical margins (27% versus 17%; *P* = 0.01) than GG1 patients (Table 1). None of the GG1 or GG2− patients had metastasis at lymph node dissection.

**Table 1:** Clinicopathological characteristics of prostate cancer patients with Grade Group 1 (GG1), Grade Group 2 without cribriform architecture (GG2−) and Grade Group 2 with cribriform architecture (GG2+)

	GG1 (*N* = 207)	GG2− (*N* = 192)	*P*‐value*	GG2+ (*N* = 228)
Age (years), mean (median; IQR)	62.5 (63.2; 59.8–66.7)	63.2 (64.0; 59.2–68.1)	0.26	64.2 (64.9; 60.3–67.9)
PSA (ng/ml), mean (median; IQR)	7.0 (6.3; 4.0–9.2)	9.4 (7.7; 5.4–10.5)	<0.001	12.2 (8.3; 6.3–14.0)
pT stage, *n* (%)				
T2	185 (89)	124 (64)	<0.001	110 (48)
T3a	20 (10)	63 (33)		90 (40)
T3b	2 (1)	5 (3)		28 (12)
Gleason pattern 4 (%),mean (median; IQR)	0.6 (0; 0–0)	18 (15; 10–25)	<0.001	24 (20; 15–30)
Invasive cribriform carcinoma, *n* (%)	5 (2)^†^	0	0.03	204 (90)
Intraductal carcinoma, *n* (%)	4 (2)†	0	0.05	103 (45)
Tertiary Gleason pattern 5, n (%)	1 (0.5)	18 (9)	<0.001	31 (14)
Positive surgical margin status, *n* (%)	35 (17)	52 (27)	0.014	90 (40)
Pelvic lymph node dissection, *n* (%)	134 (65)	91 (47)	<0.001	150 (66)
Lymph node metastasis	0	0	–	12 (8)
Biochemical recurrence, *n* (%)	16 (8)	29 (15)	0.02	67 (29)
Metastasis, *n* (%)	0	0	–	13 (6)
Disease‐specific death, *n* (%)	0	0	–	3 (1)

IQR, interquartile range; PSA, prostate‐specific antigen.

* *P*‐values represent statistical comparison of GG1 and GG2−.

^†^ Invasive cribriform and intraductal carcinoma as tertiary components in GG1.

### Tertiary Gleason Pattern 4 In GG1 Prostate Cancer

GG1 prostate cancer in radical prostatectomy specimens might, by definition, contain tertiary high‐grade patterns. To investigate to what extent GG2− prostate cancer differed from GG1 prostate cancer with a tertiary pattern and/or pure GG1 prostate cancer, we analysed both GG1 subgroups separately. Tertiary Gleason pattern 4 was present in 42 of 207 (20%) GG1 patients, of whom nine (4%) had cribriform architecture. Tertiary Gleason pattern 5 was present in only one (0.5%) patient. Patients with tertiary Gleason pattern 4 had higher median PSA levels (8.4 ng/ml versus 6.6 ng/ml; *P* = 0.01), more frequent extraprostatic extension (41% versus 3%; *P* < 0.001) and more positive surgical margins (43% versus 10%; *P* < 0.001) than GG1 patients without a tertiary pattern. Although GG2− patients had a higher percentage of Gleason pattern 4 (18% versus 3%; *P* < 0.001) and more often had tertiary Gleason pattern 5 (9% versus 0.5%; *P* = 0.04) than GG1 patients with tertiary Gleason pattern 4, PSA levels (9.4 ng/ml versus 8.4 ng/ml; *P* = 0.4) and extraprostatic extension (36% versus 41%; *P* = 0.7) were not statistically different.

### Clinical Outcome

**Figure 1:**
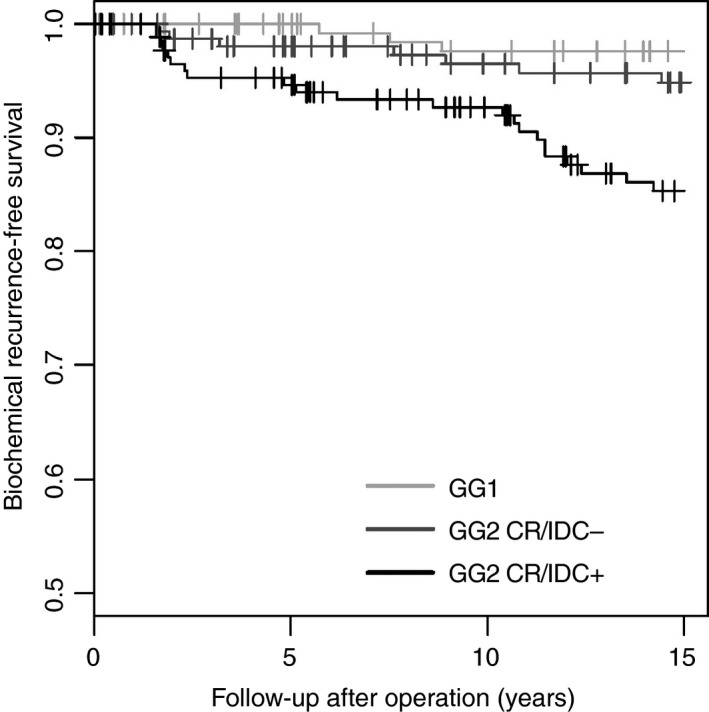
Kaplan–Meier curves for biochemical recurrence‐free survival in Grade Group 1 (GG1) and Grade Group 2 prostate cancer patients with (GG2 CR/IDC+) and without (GG2 CR/IDC−) cribriform architecture. Log rank *P* < 0.001.

The median follow‐up of the entire cohort was 59.6 months (IQR 17.5–113.9 months). Biochemical recurrence occurred in 112 (18%) patients after a median of 29.9 months (IQR 11.6–55.5 months). GG2− patients had shorter biochemical recurrence‐free survival than GG1 patients, and those with cribriform and/or intraductal carcinoma had the worst survival outcome (overall log rank, *P* < 0.001; Figure 1). The biochemical recurrence‐free survival rates of GG1 patients with tertiary Gleason pattern 4 and GG2− patients were similar (log rank, *P* = 0.4).

In univariate Cox regression analysis, PSA level [hazard ratio (HR) 1.03, 95% confidence interval (CI) 1.02–1.04; *P* < 0.001], pT stage (HR 2.7, 95% CI 1.8–4.1; *P* < 0.001), percentage of Gleason pattern 4 (HR 1.03, 95% CI 1.02–1.04; *P* < 0.001), tertiary Gleason pattern 5 (HR 2.4, 95% CI 1.4–4.2; *P* = 0.002), positive surgical margins (HR 3.5, 95% CI 2.4–5.1; *P* < 0.001), positive lymph nodes (HR 20.1, 95% CI 9.8–41.5; *P* < 0.001) and Grade Groups were all significantly associated with biochemical recurrence‐free survival (Table 2). In multivariable analysis, GG2+ (HR 3.0, 95% CI 1.4–6.3; *P* = 0.004), pT3 stage (HR 1.6, 95% CI 1.0–2.4; *P* = 0.05), positive surgical margins (HR 2.3, 95% CI 1.6–3.5; *P* < 0.001) and positive lymph nodes (HR 7.2, 95% CI 3.0–17.2; *P* < 0.001) had independent predictive value for biochemical recurrence‐free survival. Although GG2− patients had shorter biochemical recurrence‐free survival (HR 1.9, 95% CI 0.9–3.8) than GG1 patients, this did not meet conventional measures of significance (*P* = 0.08) in multivariate analysis.

**Table 2:** Cox regression analysis of biochemical recurrence‐free survival in patients with Grade Group 1 (GG1) and Grade Group 2 prostate cancer with (GG2+) and without (GG2−) cribriform architecture

	Univariate analysis			Multivariable analysis		
	HR	95% CI	*P*‐value	HR	95% CI	*P*‐value
Age	0.99	0.96–1.02	0.58	0.99	0.96–1.02	0.52
PSA	1.03	1.02–1.04	<0.001	1.01	1.00–1.02	0.13
pT stage						
T2	Ref.			Ref.		
T3a	2.7	1.8–4.1	<0.001	1.6	1.0–2.4	0.05
T3b	9.9	5.8–16.9	<0.001	2.7	1.4–5.4	0.005
Percentage Gleason 4	1.03	1.02–1.04	<0.001	1.0	1.0–1.02	0.69
Tertiary Gleason pattern 5	2.4	1.4–4.2	0.002	1.4	0.7–2.6	0.34
Positive surgical margin status	3.5	2.4–5.1	<0.001	2.3	1.6–3.5	<0.001
Lymph node metastasis	20.1	9.8–41.5	<0.001	7.2	3.0–17.2	<0.001
Grade Group						
GG1	Ref.			Ref.		
GG2−	2.7	1.4–4.9	0.002	1.9	0.9–3.8	0.08
GG2+	6.2	3.6–10.7	<0.001	3.0	1.4–6.3	0.004

CI, confidence interval; HR, hazard ratio; PSA, prostate‐specific antigen.

During follow‐up, 13 (6%) GG2+ patients developed distant metastases, of whom three had positive lymph nodes at the time of radical prostatectomy. Whereas, in total, 22 (10%) GG2+ patients had developed either lymph node or distant metastases, no metastases were identified in any GG2− patients or GG1 patients at the time of surgery or during follow‐up. Three patients died from prostate cancer, and all of them were GG2+ patients.

## Discussion

During the last decade, various studies demonstrated that GG2 patients with invasive cribriform and/or intraductal carcinoma have worse disease outcomes than those without.^8^, ^9^, ^14^, ^16^, ^28^, ^29^ Although it is generally accepted that GG2 patients have more aggressive disease than GG1 patients, it is unclear whether this is still the case when those with aggressive cribriform pathology are excluded. In this study, we found that 46% of patients with GG2 prostate cancer at the time of radical prostatectomy had neither invasive cribriform nor intraductal carcinoma. These patients had significantly higher PSA levels, pT stage, and positive surgical margin rates, and shorter biochemical recurrence‐free survival, than GG1 patients. However, none of the 399 GG1 or GG2− patients had metastasis at the time of surgery or during follow‐up, whereas metastases were identified in 10% of GG2+ patients. These findings indicate that invasive cribriform and/or intraductal carcinoma might have the most impact on metastatic disease progression.

Although both invasive cribriform and intraductal carcinoma are pathological features associated with tumour aggressiveness, it is not yet clear how to incorporate these parameters in clinical risk stratification. For instance, Iczkowski *et al*. proposed modifying the current Grade Groups 2 to 4 by reporting the presence of invasive cribriform and intraductal carcinoma denoted with a ‘C’, which would increase the number of risk groups from five to eight.^30^ However, it is not yet evident whether clinically relevant differences exist between these subgroups or whether they partially overlap. Previously, our group found that biopsy GG2− patients had similar biochemical recurrence rates and disease‐specific survival to those of GG1 patients.^15^, ^16^, ^17^ In the current study, we demonstrate that GG2− prostate cancer is associated with significantly worse clinicopathological characteristics and outcome than GG1 prostate cancer. These findings on radical prostatectomy specimens differ slightly from those of our previous study on biopsy specimens.^17^ This might be explained by biopsy sampling artefacts, as upgrading occurs in up to 40% of biopsy GG1 and GG2 patients.^1^, ^31^ Furthermore, recent studies have indicated that biopsies have a moderate sensitivity of 43–47% for detecting cribriform architecture.^21^, ^22^ Despite moderate concordance of growth patterns between biopsy and radical prostatectomy specimens, incorporation of cribriform architecture into the Grade Groups has better discriminative value for disease‐specific survival and metastasis‐free survival.^20^ In biopsies, we previously demonstrated that subtraction of one point of the Grade Group if no cribriform architecture was present was a simple and valuable modification of the current prostate cancer grading scheme.20

Metastasis‐free survival rates of GG2− and GG1 patients were similar in both biopsy and radical prostatectomy studies.^17^ GG1 prostate cancer at the time of radical prostatectomy is known to have a very low if any risk of metastasis and disease‐specific death.^5^, ^32^, ^33^, ^34^, ^35^, ^36^, ^37^, ^38^, ^39^ As no metastases were identified in pelvic lymph node dissection or during follow‐up of GG2− patients, this population also seems to have a low risk of metastatic progression. This indicates that invasive cribriform and intraductal carcinoma, in particular, might have an impact on the biological potential for metastatic disease to develop. In contrast, postoperative biochemical recurrence‐free survival is also related to tumour volume parameters and surgical technique, which do not necessarily reflect biological derangement caused by the disease. Cribriform architecture has been associated with genomic instability and has been clonally related to lymph node metastasis, which might provide a rationale for its aggressive biological behaviour.^40^, ^41^, ^42^, ^43^

Forty‐two of 207 (20%) GG1 patients had tertiary Gleason pattern 4. These patients had worse clinicopathological features than pure GG1 patients, and were more similar to GG2− patients. This finding is in line with those of others reporting on the clinical relevance of tertiary patterns, and underlines the importance of reporting them.^44^, ^45^, ^46^, ^47^, ^48^, ^49^, ^50^, ^51^, ^52^, ^53^

The strong point of this study is the detailed histological evaluation of the radical prostatectomy specimens, including recently identified clinically relevant pathological parameters. Limitations of this retrospective investigation are its relatively low number of patients and its limited median follow‐up time of 59.6 months. The identification of small differences in metastasis‐free survival would require a large number of low‐risk patients with long‐term follow‐up. Finally, although we made significant efforts to differentiate intraductal and invasive cribriform carcinoma, this distinction might be impossible in some cases, even with the use of immunohistochemistry.

In conclusion, patients with GG2− prostate cancer at the time of radical prostatectomy have more advanced disease and shorter biochemical recurrence‐free survival than GG1 patients. However, both groups have a very low risk of developing metastatic disease.

## Conflicts of interest

The authors declare no conflicts of interest.

## Author contributions

E. Hollemans and Geert J.L.H. van Leenders had full access to all the data in the study and take responsibility for the integrity of the data and the accuracy of the data analysis. Study concept and design: vans Leenders. Acquisition of pathology data: Hollemans, Verhoef. Supply of clinical data: Bangma, Rietbergen, Roobol, Helleman. Analysis and interpretation of data: Hollemans, Verhoef, van Leenders. Drafting of the manuscript: Hollemans, van Leenders. Critical revision of the manuscript for important intellectual content: Hollemans, Verhoef, Bangma, Rietbergen, Roobol, Helleman, van Leenders. Statistical analysis: Hollemans, van Leenders. Obtaining funding: None. Administrative, technical, or material support: Hollemans, Verhoef. Supervision: van Leenders.
